# Comment on “Evolutionary transitions between beneficial and phytopathogenic *Rhodococcus* challenge disease management”

**DOI:** 10.7554/eLife.35272

**Published:** 2018-05-08

**Authors:** Jennifer J Randall, Rio A Stamler, Craig E Kallsen, Elizabeth J Fichtner, Richard J Heerema, Peter Cooke, Isolde Francis

**Affiliations:** 1 Entomology, Plant Pathology, and Weed ScienceNew Mexico State UniversityLas CrucesUnited States; 2 University of California, Cooperative ExtensionBakersfieldUnited States; 3 University of California, Cooperative ExtensionTulareUnited States; 4 Department of Plant and Environmental SciencesNew Mexico State UniversityLas CrucesUnited States; 5 Core University Research Resource LaboratoryNew Mexico State UniversityLas CrucesUnited States; 6 Department of BiologyCalifornia State UniversityBakersfieldUnited States; University of ChicagoUnited States

**Keywords:** pistachio, *Rhodococcus fascians*, Pistachio Bushy Top Syndrome, virulence factors, Other

## Abstract

We would like to address a number of concerns regarding this paper (Savory et al., 2017)

## Plant growth promotion by the *Rhodococcus* pistachio bushy top syndrome (PBTS) isolates

While the analysis by Savory et al. (2017) of *Rhodococcus fascians* isolates that are found to cause leafy gall disease on ornamental plants is noteworthy, the data presented do not unambiguously demonstrate plant growth promotion by PBTS *Rhodococcus* isolates. In contrast, the data presented in Savory et al. (2017) (Figure 7B and Figure 7—figure supplement 1) indicate that, upon inoculation, the *Nicotiana benthamiana* roots were stunted in comparison to the roots of control plants. PBTS *Rhodococcus* 2 caused significant root stunting at an inoculum level of only 2.5 × 10^6^ CFU (Figure 7B), 10^3^ fold lower than the standard dose used throughout the paper. In orchard systems, inadequate root development affects tree anchorage in soils and influences aboveground tree and canopy development. For these reasons, the data presented by Savory et al. do not support the assertion that PBTS *Rhodococcus* isolates are plant growth promoting.

## Potential misdiagnosis of an epidemic

Savory et al. claim to have ‘uncovered a misdiagnosed epidemic’ that has ‘perpetuated the unnecessary removal of trees and has exacerbated economic losses in the pistachio industry’. The trees that exhibited Pistachio Bushy Top Syndrome (PBTS) were removed due to their irregular growth and development, and their high percentage of grafting failure (>70%; Stamler et al., 2015a). Those rootstocks that did successfully accept a scion bud developed bark cracking along the bud/graft union that weakened the tree. Pistachio ranch owners and managers of affected orchards concluded that tree removal was the only economically feasible option well before Stamler et al., 2015a was published. An ongoing study has demonstrated that PBTS trees entering maturity exhibit a 73% reduction in yield and lower nut quality in comparison to asymptomatic trees of the same age in the same orchard (Fichtner et al., 2018). Ranch managers opted for the more expensive hand-harvest, as opposed to mechanical harvest of symptomatic trees, due to the risk of breakage at the cracked graft union. Three of the authors on Stamler et al., 2015a are pistachio horticulturists with close connections to the pistachio industry. We are personal witnesses to the fact that the statements regarding tree removal in California, Arizona and New Mexico are inaccurate (Savory et al., 2017). The majority of PBTS symptomatic trees were removed from orchards a year before Stamler et al., 2015a was published (Northcutt, 2015; Fichtner et al., 2015). Hence, the claim that the trees were removed due to a misdiagnosis is erroneous, unjustly discredits the research team involved in the diagnosis, and insults the pistachio growers who decided to remove trees.

## Presence of virulence genes

Savory et al. state that 'contrary to previous findings, there is no evidence to support the diagnosis that *Rhodococcus* without a virulence plasmid are responsible for an unusual growth problem that has plagued the pistachio industry.’ It was never claimed that PBTS was caused by *Rhodococcus* isolates lacking virulence genes (Stamler et al., 2015a). On the contrary, the isolates utilized for establishing Koch’s postulates were shown to *have* virulence genes (*fasD* and *fasA*) using PCR amplification and direct sequencing (Stamler et al., 2015a). Since previous work on *R. fascians* isolates from ornamental plants has shown that the presence of virulence genes is required for pathogenicity (Desomer et al., 1988; Crespi et al., 1992; Eason et al., 1995; Eason et al., 1996; Stange et al., 1996; Nikolaeva et al., 2009), we agree that PBTS isolates without these described virulence genes are likely non-pathogenic.

## Subpopulations and loss of detectable virulence genes in PBTS *Rhodococcus* isolates when culturing on synthetic media

Savory et al. use a ‘Genome Announcement’ (Stamler et al., 2016) as evidence for the lack of virulence genes in PBTS1 and PBTS2, although the title of the cited paper clearly stated that the information provided concerned draft sequences of PBTS1 and PBTS2, thus recognizing that not all of the genetic information was obtained (i.e., *fasD*, *fasA*, and *attA*). Our unpublished work has shown that these specific *Rhodococcus* isolates obtained from UCB-1 pistachio trees and grown on synthetic media contain a subpopulation of bacterial cells with virulence genes (*fasD* and *fasA*). After subculturing the bacteria only twice on synthetic media, these virulence genes become undetectable by PCR. Thus, when sequencing these subcultured isolates using the PacBio platform, the virulence genes (originally detected using PCR amplification) were no longer detected (Stamler et al., 2016). Plasmid instability and loss of virulence are recognized in several bacterial species and were also described for leafy gall-inducing *R. fascians* isolates. Sabart et al., 1986 demonstrated that *R. fascians (*previously named *Corynebacterium fascians)* lost virulence within several generations due to growing conditions, and Nikolaeva et al., 2009 showed instability of virulence due to plasmid loss in certain *R. fascians* isolates.

## High plasmid instability in *R. fascians*

Savory et al. state that Nikolaeva et al., 2009 used the wrong molecular tools to evaluate pathogenicity, but these authors showed that for some isolates, individual *fas* positive colonies gave rise to *fas* positive and *fas* negative bacteria, very effectively ruling out the possibility that these findings were caused by genetically different lineages.

## Poor growth of the control pistachio UCB-1 trees

**Figure 1.**
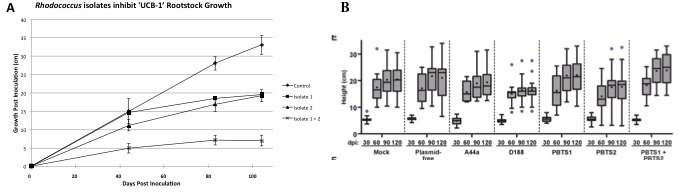
Comparison of pistachio ‘UCB-1’ clonal tree assays versus ‘UCB-1’ seedling tree assays. (**A**) Clonal UCB-1 trees, transitioned out of tissue culture eight weeks prior to inoculation with *Rhodococcus* isolates 1 and 2. Four treatments were performed with 16 plants per treatment for each trial. The control plants were mock-inoculated with buffer. Three inoculated treatments included bacterial suspensions of *Rhodococcus* isolate 1, *Rhodococcus* isolate 2, and both *Rhodococcus* isolate 1 and isolate 2. Growth curves showing height of UCB-1 plants at multiple time points after inoculation. Error bars are standard error (*n* = 16). (**B**) Seedling UCB-1 trees inoculated with *Rhodococcus*. Reprinted from Savory et al., 2017. *eLife* **6**:e30925.

The work by Savory et al. indicates that *R. fascians* and PBTS *Rhodococcus* 1 and 2 were inoculated on UCB-1 seedlings and no effects were observed (Figure 7—figure supplement 1; panel E). Savory et al. provided no details in regards to the germination, size of seedlings, or growth conditions. In our work, micropropagated, clonal UCB-1 plants were used to mimic the propagation type of affected PBTS trees in the field while minimizing the potential influence of host variability on symptom expression (Stamler et al., 2015a). Based on the data provided in Savory et al. (Figure 7—figure supplement 1; panel E), all of the UCB-1 pistachio seedlings surprisingly exhibited little to no growth after 60 days. In contrast, the mock-inoculated clonal trees used by Stamler et al., 2015a continued to grow rapidly for more than 100 days, and it was nearly 80 days post-inoculation before statistically significant differences in height were observed between inoculated and mock-inoculated trees (Figure 1). The poor growth of mock-inoculated UCB-1 seedlings (Savory et al., 2017) suggests that experimental conditions were not conducive to healthy pistachio growth, thus rendering the control trees unsuitable as a basis of comparison.

## Misrepresentation of previous work

- ‘There was a targeted search for *Rhodococcus*’ (referring to Stamler et al., 2015a, 2015b). This is not true, as many potential causative agents were evaluated.
- ‘Bacteria were inexplicably cultured from asymptomatic leaves distal to symptomatic tissues’. If Savory et al. are implying that *R. fascians* can only be cultured from symptomatic tissues, this has been known for decades (Lacey, 1939; Cornelis et al., 2001).
- ‘Some of the results of the molecular detection were clearly artifacts,’ referring to Stamler et al., 2015a. Savory et al. support their statement by stating that the published *fasD* fragments are identical in sequence, yet they fail to mention that the *fasD* gene over its entire length is identical among most of the ornamental isolates (Creason et al., 2014).
- By stating, ‘We recognize the insurmountable challenge in showing that there is no possibility that pathogenic *Rhodococcus* causes pistachio bushy top syndrome,’ Savory et al. admit to the fact that their data did not show a misdiagnosis. We remain confident in our published results.
